# Cardiac Tamponade Secondary to a Giant Pleuropericardial Cyst in a Child: A Surgical Case Report from Sub-Saharan Africa

**DOI:** 10.1055/a-2655-3348

**Published:** 2025-07-28

**Authors:** Abdel Kémal Bori Bata, Yacoubou Imorou-Souaibou, Ahmad Ibrahim, Désiré Nékoua, Joseph Adoco, Arnaud Sonou

**Affiliations:** 1 University Visceral Surgery Clinic (CNHU-HKM), Faculty of Health Sciences, University of Abomey-Calavi, Cotonou, Benin; 2 University Cardiology Clinic (CNHU-HKM), Faculty of Health Sciences, University of Abomey-Calavi, Cotonou, Benin; 3 University Hospital Center of the Abomey-Calavi/So-Ava Zone (CHUZ/AS), Faculty of Health Sciences, University of Abomey-Calavi, Cotonou, Benin; 4 University Cardiology Clinic (CNHU-HKM), Faculty of Health Sciences, University of Abomey-Calavi, Cotonou, Benin; 5 Multipurpose University Clinic of Anesthesia and Resuscitation (CNHU-HKM), Faculty of Health Sciences, University of Abomey-Calavi, Cotonou, Benin; 6 University Cardiology Clinic (CNHU-HKM), Faculty of Health Sciences, University of Abomey-Calavi, Cotonou, Benin

**Keywords:** pleuropericardial cyst, child, surgical resection, sub-Saharan Africa

## Abstract

Pleuropericardial cysts are rare mediastinal tumors with variable, often severe, clinical presentations in children, occasionally requiring urgent intervention. We report the case of a previously healthy 14-year-old male who was admitted with signs of severe congestive heart failure and clinical evidence of cardiac tamponade. Transthoracic echocardiography and thoracic CT scan confirmed the presence of a compressive mediastinal cystic mass. The patient underwent emergency surgical resection via median sternotomy. Histopathological examination confirmed a benign pericardial cyst. Postoperative recovery was uneventful, and no recurrence was observed after 2 years of follow-up.

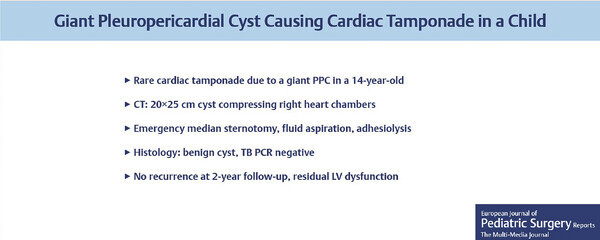

## Introduction

Pleuropericardial cysts (PPCs) are rare congenital mediastinal anomalies in children, accounting for 5 to 10% of mediastinal tumors, with an incidence of approximately 1 per 100,000 individuals. 1 2 Although they can occur at any age, most reported cases involve middle-aged adults (40–50 years), and pediatric occurrences remain uncommon. 3 PPCs are asymptomatic in 75% of patients. However, two-thirds of pediatric cases present with symptoms due to compression or invasion of adjacent structures. 1 4 We report a favorable outcome in a teenager surgically treated for a symptomatic PPC.

## Case Report

A 14-year-old male adolescent presented with a 2-month history of progressively worsening exertional dyspnea, lower limb edema, and intermittent fever over the previous month. On admission, he exhibited signs of severe global heart failure with clinical features of cardiac tamponade.

**Fig. 1:**
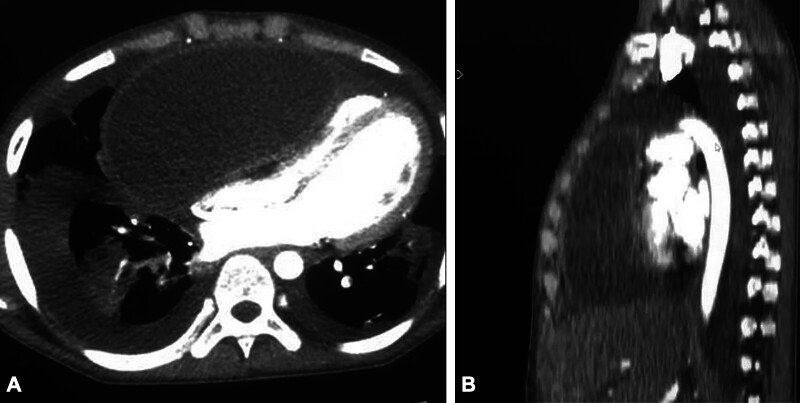
( **A** ) Axial CT scan showing the pleuropericardial cyst (PPC) compressing the right heart chambers. ( **B** ) Sagittal contrast-enhanced CT scan showing a large PPC anteriorly displacing and compressing the right atrium and ventricle.

Chest radiography revealed mediastinal widening with a cardiothoracic index of 0.72 and bilateral basal pleuropneumonia. Transthoracic echocardiography showed right chamber compression with impaired diastolic filling due to a hypoechoic mass, along with a severely depressed left ventricular ejection fraction (35%). Thoracic CTA revealed a large anterior mediastinal cystic mass (20 × 25 cm) compressing the right cardiac chambers, displacing the heart leftward, and causing significant cavosuprarenal reflux ( Fig. 1 ). Additional findings included bilateral lobar pulmonary embolism with small infarctions, moderate bilateral pleural and passive parenchymal atelectasis, and mediastinal lymphadenopathy.

**Fig. 2:**
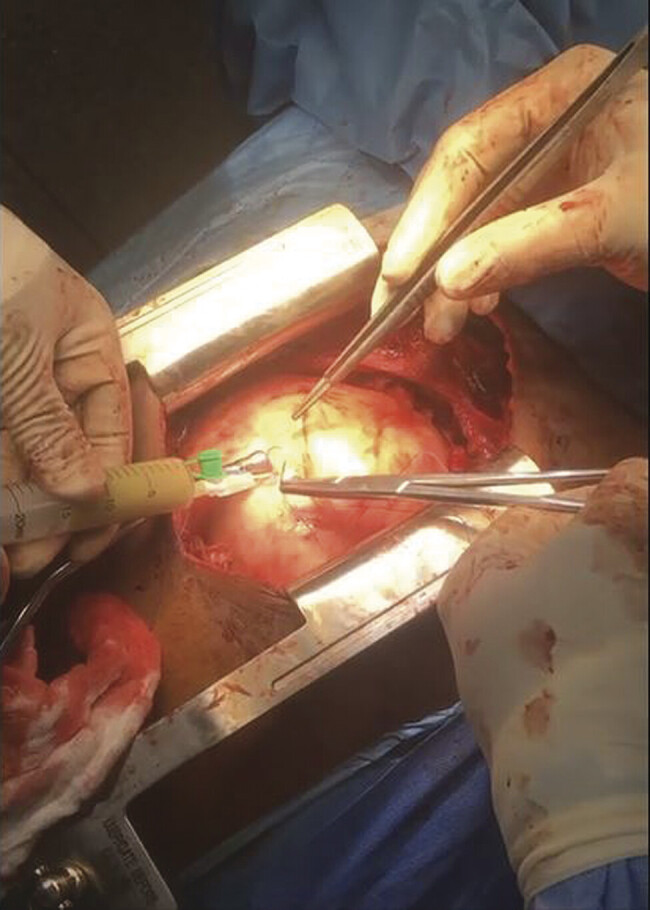
Aspiration of whitish cystic fluid during surgical resection of the PPC. PPC. pleuropericardial cyst.

Emergency surgery was performed via full median sternotomy. Intraoperatively, the cystic wall appeared white and adherent to the diaphragm, both pleurae, and the anterior heart surface. Due to the failure of en bloc resection, aspiration of 500 mL of whitish fluid was performed, followed by laborious adhesiolysis ( Fig. 2 ). Histopathological analysis confirmed a benign pericardial cyst. PCR testing for *Mycobacterium tuberculosis* was negative. Cytological analysis revealed rare leukocytes, and cultures were sterile.

Adjuvant treatment included anticoagulation and heart failure therapy (ACE inhibitors, β-blockers, aldosterone antagonists). Clinical and hemodynamic improvement was noted postoperatively, with persistent fever due to a coagulase-negative *Staphylococcus* bacteremia, successfully managed with targeted antibiotics.

At 2-year follow-up, echocardiography revealed no recurrence and a persistent left ventricular dysfunction.

## Discussion

PPCs are rare benign mediastinal tumors, particularly in children under 18 years old, with fewer than 20 cases reported in the literature. 5 They are usually congenital, arising from failed fusion of the primitive pericardial lacunae or abnormal folding of embryonic pleura during pericardial development. 6 Symptoms range from asymptomatic presentation to exertional dyspnea, chest pain, or cough, depending on the compression of mediastinal structures. Severe complications include right ventricular outflow obstruction, pulmonary stenosis, cardiac tamponade, partial erosion of the superior vena cava, and congestive heart failure. 7 8 In this case, the clinical picture was dominated by tamponade physiology and global heart failure.

Imaging plays a crucial role in diagnosing pediatric mediastinal masses. Chest radiography is the first-line modality, while CT is the preferred imaging technique to define cyst origin, morphology, and anatomical relationships. MRI is increasingly used for lesion characterization. 9 PPCs are typically located in the right (77%) or left (22%) cardiophrenic angles. 1 6 In our patient, the cyst was located in the right anterolateral mediastinum.

Asymptomatic PPCs may be managed conservatively with imaging surveillance. However, surgical excision is recommended for symptomatic, enlarging, atypical, or diagnostically uncertain cysts or in athletes at risk of rupture. 10 While traditionally performed via thoracotomy or sternotomy, video-assisted thoracoscopic surgery (VATS) is now favored due to PPCs' typical characteristics (thin-walled, poorly vascularized, loosely adherent). 10 Image-guided percutaneous aspiration is an alternative option. 8 In our case, the choice of full median sternotomy was guided by the cyst's diaphragmatic adhesion, the patient's hemodynamic instability, and the limited availability of minimally invasive tools in sub-Saharan Africa. At 2-year follow-up, the absence of recurrence confirmed the effectiveness of this approach. Literature reports a 33% recurrence rate with percutaneous aspiration. 10

Histopathology remains essential for diagnosis and exclusion of differential diagnoses, such as pericardial lymphangioma. 8 Although the true incidence of tuberculous PPCs is unknown, a few cases of tuberculous origin have been reported in the literature. 11 Given that our country is considered a tuberculosis-endemic area, a microbiological assessment for *Mycobacterium tuberculosis* was conducted, and the results were negative. This case underscores the potential severity of PPCs and the importance of timely surgical management, even for lesions that are typically considered benign.

## Conclusion

PPCs are rare, benign mediastinal tumors in children. Although often asymptomatic, they can cause life-threatening complications such as tamponade. Total surgical resection via median sternotomy proved effective in this case, with no recurrence at 2-year follow-up.

## Notes

Importance for a Pediatric Surgeon This case highlights the importance of recognizing pleuropericardial cysts as a rare but life-threatening cause of cardiac tamponade in children. It underscores the need for timely surgical intervention, even in resource-limited settings, to ensure favorable outcomes.
